# Embodiment and Presence in Virtual Reality After Stroke. A Comparative Study With Healthy Subjects

**DOI:** 10.3389/fneur.2019.01061

**Published:** 2019-10-10

**Authors:** Adrián Borrego, Jorge Latorre, Mariano Alcañiz, Roberto Llorens

**Affiliations:** ^1^Neurorehabilitation and Brain Research Group, Instituto de Investigación e Innovación en Bioingeniería, Universitat Politècnica de València, Valencia, Spain; ^2^NEURORHB-Servicio de Neurorrehabilitación de Hospitales Vithas, Valencia, Spain

**Keywords:** embodiment, body-ownership, self-location, agency, presence, stroke, virtual reality, immersion

## Abstract

The ability of virtual reality (VR) to recreate controlled, immersive, and interactive environments that provide intensive and customized exercises has motivated its therapeutic use after stroke. Interaction and bodily presence in VR-based interventions is usually mediated through virtual selves, which synchronously represent body movements or responses to events on external input devices. Embodied self-representations in the virtual world not only provide an anchor for visuomotor tasks, but their morphologies can have behavioral implications. While research has focused on the underlying subjective mechanisms of exposure to VR on healthy individuals, the transference of these findings to individuals with stroke is not evident and remains unexplored, which could affect the experience and, ultimately, the clinical effectiveness of neurorehabilitation interventions. This study determined and compared the sense of embodiment and presence elicited by a virtual environment under different perspectives and levels of immersion in healthy subjects and individuals with stroke. Forty-six healthy subjects and 32 individuals with stroke embodied a gender-matched neutral avatar in a virtual environment that was displayed in a first-person perspective with a head-mounted display and in a third-person perspective with a screen, and the participants were asked to interact in a virtual task for 10 min under each condition in counterbalanced order, and to complete two questionnaires about the sense of embodiment and presence experienced during the interaction. The sense of body-ownership, self-location, and presence were more vividly experienced in a first-person than in a third-person perspective by both healthy subjects (*p* < 0.001, $\eta_{\text{p}}^{2}$ = 0.212; *p* = 0.005, $\eta_{\text{p}}^{2}$ = 0.101; *p* = 0.001, $\eta_{\text{p}}^{2}$ = 0.401, respectively) and individuals with stroke (*p* = 0.019, $\eta_{\text{p}}^{2}$ = 0.070; *p* = 0.001, $\eta_{\text{p}}^{2}$ = 0.135; *p* = 0.014, $\eta_{\text{p}}^{2}$ = 0.077, respectively). In contrast, no agency perspective-related differences were found in any group. All measures were consistently higher for healthy controls than for individuals with stroke, but differences between groups only reached statistical significance in presence under the first-person condition (*p* < 0.010, η${}_{\text{p}}^{2}$ = 0.084). In spite of these differences, the participants experienced a vivid sense of embodiment and presence in almost all conditions. These results provide first evidence that, although less intensively, embodiment and presence are similarly experienced by individuals who have suffered a stroke and by healthy individuals, which could support the vividness of their experience and, consequently, the effectiveness of VR-based interventions.

## Introduction

Classical definitions of embodiment have resorted to the concepts of corporeal awareness (1), bodily self-consciousness (2), and the sense of one's own body (3). Embodiment, however, is a complex multi-component phenomenon that could be better described as the representation of an element within the body schema (4), the mental representation of body parts and reachable space, which effectively extends or displaces the normal area of influence of the body parts by real or artificial body parts, habitually used tools, or prostheses (5). Although its neural mechanisms are unclear, it is hypothesized that embodiment operates both via automatic bottom-up and potentially conscious top-down processes to permit the establishment of sensorimotor maps of one's body parts with respect to one's body (5). Previous research has identified different constitutive components of embodiment, including body-ownership, self-location, and agency (5). An understanding of their dissociation is, however, uncertain (6). Body-ownership can be defined as the sense that the body that one inhabits is one's own. Self-location alludes to the sense of being in the place where one's body is. Agency refers to the sense that one can move and control one's own body.

Experiments on multisensory and/or sensorimotor stimulation have allowed remarkable modulations of the perceived body to be performed, with respect to its natural configuration, which are known as body illusions (7). Body illusions have enabled the investigation of the embodiment subcomponents separately, with a particular emphasis on body-ownership. Because body-ownership should be continuous and omnipresent (8), synchronous visuotactile stimulation has been proven to be sufficient to induce this sense not only over rubber hands (9) or feet (10), but also over mannequins (11), in the absence of agency. In contrast, only experiments involving self-triggered actions, fired by efferent signals, have elicited agency (12). Virtual reality (VR) is a paradigmatic case of the former, as it provides multisensory stimulation while allowing real-time user interaction (13). Previous experimentation employing VR has successfully induced embodiment over virtual body parts (14, 15) and entire virtual bodies (16). Importantly, embodying virtual selves may not only affect the body schema, but could also modulate perception (17) and behavior, according to the physical characteristics of the incarnated avatar (18, 19).

Experience in VR is likewise strongly modulated by the sense of presence, which is the sense of being in the virtual environment (VE) (20) or, in other words, the sense of existing inside it (21). Similarly to embodiment, presence is a multi-component construct (22). Both user characteristics (either demographical, psychological, or clinical) and media characteristics (content or form, also known as immersion) contribute to the experience (22). However, the interaction between presence and immersion, the extent to which VR is capable of delivering an illusion of reality to the human senses (13), is not obvious. Nevertheless, a greater sense of presence is expected for higher levels of immersion, provided that other characteristics of the experience remain unchanged (22).

Although it seems reasonable that being in a specific environment can impact the sense of having, moving, and being in a body, and vice versa, research has focused on each construct individually and, consequently, interactions between embodiment and presence remain underexplored. A preliminary uncontrolled study using consumer HMDs attempted to find interactions between embodiment and presence by modulating the existence of a static avatar in the VE, which was supposed to be embodied by the participants (23). The experiment did not find connections between the investigated constructs because, among other possible reasons, it failed to elicit embodiment over the avatar, which was not able to reproduce the participants' movements. Analogously, a study with an augmented reality-based mirror found no connections between embodiment and presence because, in this case, it failed to promote presence in the VE (24). Another experiment by the same group, however, was successful at promoting high levels of embodiment and presence in a mixed reality environment, but interactions between constructs were not discussed (25). The only true attempt made to disentangle this interrelation suggested that perspective influences body-ownership and self-location over a virtual avatar, but not presence nor agency (26), which contradicts previous reports on presence (27–29).

The ability of VR to recreate controlled, immersive, and interactive environments that engage participants in intensive and customized exercises, has motivated its use in different neurological populations, especially stroke. VR-based exercises provide goal-directed tasks that are accomplished in the virtual world by the actions of virtual selves, which are controlled by the participants (30). Users usually experience the virtual world either from a first-person (egocentric) perspective, using a head-mounted display (HMD), or from a third-person (allocentric) perspective, displayed on a screen. In motor interventions, interaction is usually facilitated by body movements, which are transferred to a virtual avatar that mimics the actions in the virtual world (31–33). Although an increasing number of studies show the potential of VR-based interventions on motor function after stroke (34, 35), with a special emphasis on balance (36) and upper limb (37), little is known about how VR experiences are mediated in this population. On the contrary, all our insights into embodiment and presence have been provided by studies involving healthy subjects, predominantly young adults (38–41). The scant existing literature suggests that the ability to sense presence after stroke may be preserved (42), but there have been no previous reports on the ability to embody virtual selves. A few reports on body illusions in the real world, involving individuals with stroke, have shown contradictory results (43, 44).

While a significant body of research has focused on the underlying subjective mechanisms of exposure to VR on healthy individuals, the transference of these findings to individuals with stroke is not evident and remains unexplored. The importance of investigating such mechanisms in individuals after stroke is that they may influence the experience and performance in the VE, which, ultimately, could affect the clinical effectiveness of neurorehabilitation interventions. Thus, the hypotheses of this study were: first, that healthy subjects can experience a vivid sense of embodiment and presence after interaction with a virtual task that would vary with the level of immersion and spatial representation, in accordance with the findings of previous studies; and second, that the elicited experience and variation would be analogously reproduced in a sample of individuals with stroke. Hence, the objectives of this study were to determine and compare perceived embodiment and presence, under different conditions of immersion and spatial representation, in samples of healthy subjects and individuals with stroke.

## Methods

### Participants

A convenience and representative sample of healthy subjects and individuals with stroke was recruited from the staff and outpatient unit of the neurorehabilitation service of Vithas Hospital Valencia al Mar (València, Spain).

Healthy subjects, with no know musculoskeletal or psychological impairment, and matched ages and genders to those of the stroke group, were recruited. Individuals with stroke were included in the study if they had the ability to understand and interact with a VR-based task. Specifically, the exclusion criteria applied to the stroke group included: first, severe cognitive impairment, as defined by scores below 23 in the Mini-Mental State Examination (45); second, an inability to follow instructions, as defined by scores below 45 in the receptive language index of the Mississippi Aphasia Screening Test (46); third, a risk of falling, as defined by scores below 45 in the Berg Balance Scale (47); fourth, visual or hearing impairment that did not allow for interaction; and finally, unilateral spatial neglect.

Forty-six healthy subjects (25 men and 21 women), with a mean age of 50.8 ± 10.9 years, agreed to participate in the study (Table 1). Thirty-two individuals with stroke (18 men and 14 women), with a mean age of 48.8 ± 11.8 years, satisfied the participation criteria and agreed to participate in the study (Table 1). These participants presented either ischemic (*n* = 25) or haemorrhagic stroke (*n* = 7), with a mean time since onset of 9.2 ± 3.0 months. Both groups were comparable in terms of age and gender.

**Table 1:** Characteristics of the participants.

	**Healthy subjects (*n* = 46)**	**Individuals with stroke (*n* = 32)**	**Significance**
Sex (*n*, %)			NS (*p* = 0.868)
Male	25 (54.3%)	18 (56.2%)	
Female	21 (45.7%)	14 (43.8%)	
Age (years)	50.8 ± 10.9	48.8 ± 11.8	NS (*p* = 0.443)
Etiology (*n*, %)			–
Ischemic stroke	–	25 (78.1%)	
Hemorrhagic stroke	–	7 (21.9%)	
Lesion side (*n*, %)			–
Left	–	22 (68.7%)	
Right	–	10 (31.3%)	
Time since injury (months)	–	9.2 ± 3.0	–
Mini-mental state examination [0–30]	–	26.4 ± 2.0	–
Mississippi aphasia screening test [0–50]	–	47.5 ± 1.6	–
Berg balance scale [0–56]	–	51.0 ± 2.9	–

*Sex, etiology, and lesion side are expressed as a percentage of the total number of participants. Age, time since injury, and scores in the clinical measures are expressed in terms of mean and standard deviation. NS, non-significant*.

Ethical approval for the study was granted by the Institutional Review Board of Vithas Hospital Valencia al Mar (TU763198CIS0/1). All participants provided written informed consent before taking part in the study.

### Instrumentation

**Figure 1:**
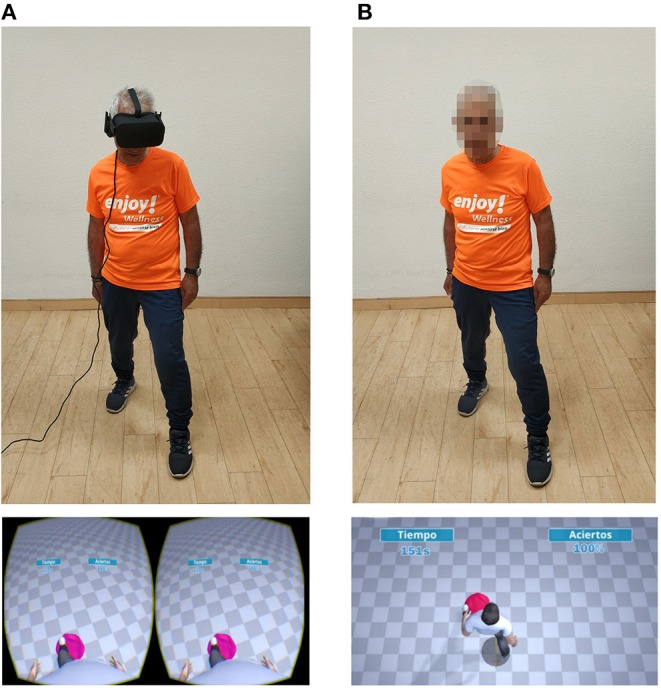
Interaction with the virtual task and virtual environment in first and third-person condition. The figure shows: **(A)** a participant interacting with the virtual task in first-person perspective (up), and the virtual environment displayed by the HMD (down), and **(B)** the same participant interacting with the virtual task in third-person perspective (up), and the virtual environment displayed by the screen **(B)**. In both conditions, the participant is squashing a pink playdough item located on the ground with his left foot.

An adaptation of an interactive VR-based stepping task, which had been previously administered to individuals with stroke for therapeutic purposes (32, 48), was used as a control task. The VE consisted of an infinite checkered floor, with a central gray circle with a diameter of 50 cm, and a gender-matched mesomorph avatar, which synchronously mimicked the participants' movements (Figure 1). Playdough-colored items (cubes, spheres, and cones), with a bounding box of 20 × 20 × 20 cm, appeared on the floor in front of the central circle. The objective of the task was to step on the items before they disappeared with the closest avatar foot, while keeping the other foot inside the central circle. In between stepping on the items, the foot used had to be moved back into the circle (32, 48). Specific animations and sound effects indicated when an item appeared, disappeared, and was squashed. Extrinsic feedback was provided during the task, with information on the number of items successfully stepped on and the remaining time (Figure 1). At the end of the task, the percentage of items stepped on was shown.

To reproduce two of the most widely used VR configurations, the VE was represented either from the avatar's egocentric point of view (first-person perspective), and displayed with a HMD, the Oculus Rift CV1 (Oculus VR, Irvine, CA), or from an allocentric point of view (third-person perspective), displayed on a 60″ LED Screen (LG, Seoul, South Korea), which was hung on a wall, with its center at ~175 cm from the floor (Figure 1). The center of the VE was defined as being at a distance of 2 m in front of the screen, which was marked on the floor. The HMD had a resolution of 2,160 × 1,200, a refresh rate of 90 Hz, and horizontal and vertical fields of view of 94° and 93°, respectively (49). The screen had a resolution of 1,920 × 1,080, a refresh rate of 60 Hz, and had approximate effective horizontal and vertical fields of view to the participants of 37° and 11°, respectively. Auditory feedback was provided by the integrated headphones in the HMD or the integrated speakers of the TV screen, as appropriate. Interaction was facilitated by a Kinect for Windows v2 (Microsoft, Redmond, WA), which was fixed under the TV at a height of 80 cm from the floor to ensure full body-tracking (50, 51). This device provided the positions of the main joints of the participants at 30 Hz. In the third-person perspective, all joints were used to animate the avatar. By way of contrast, in the first-person perspective, head rotation and acceleration were provided by the HMD (Figure 1).

A high-end computer, including an 8-core Intel® Core™ i7-4790 @3.60 GHz, 8 GB of RAM, and a NVIDIA® Geforce® GTX Titan Xp with 12 GB of GDDR5, was used to run the VE during the experiment.

### Procedure

Two experimenters were in charge of conducting the sessions and ensuring the safety and comfort of the participants. The participants, who were blind to the purpose of the experiment, were briefly introduced to the instrumentation, procedure, and task. They were then situated on the mark on the floor, looking toward the Kinect for Windows v2, and then the experiment started. All participants interacted with the VE for 10 min, in counterbalanced order, under both conditions: first-person perspective with the HMD and third-person perspective with the TV screen. After each condition, the participants were asked to evaluate their perceived sense of embodiment and presence, using two dedicated questionnaires: an adapted version of the Embodiment of Rubber Hand Questionnaire (38) and the Slater-Usoh-Steed Questionnaire (52), respectively. The adapted version of the Embodiment of Rubber Hand Questionnaire contained the same 10 items as the original version but references to a rubber hand were replaced by equivalent references to the virtual avatar. Consequently, this questionnaire assessed the extent to which the participant: can control the avatar with their movements; feels located in the same place as the avatar; and feels the body of the avatar belonged to them. The Slater-Usoh-Steed Questionnaire is a three-item Likert-scale questionnaire that evaluates: the sense of being in a VE; the extent to which a VE feels real; and the extent to which a VE is thought of as a place visited. The scores for both questionnaires ranged from 1 (strongly disagree) to 7 (strongly agree). A speech therapist was in charge of explaining the questions of the questionnaires to the participants with stroke and solving any possible doubt about their meaning.

### Data Analysis

Subcomponents of embodiment were defined, according to the original description on the questionnaire, as the average score of the first five statements (body-ownership), of the sixth to eighth statements (localization), and of the last two statements (agency) (38). Average scores >4 were considered as denoting a meaningful reflection of the vividness of the experience (38, 43).

Mixed ANOVAs were performed to determine the differences between the conditions for the individual groups (healthy and stroke) as between-subject, and perspective as within-subject, factors. The investigators performing the data analysis were blinded. The analyses were computed using SPSS for Windows® v22 (IBM®, Armonk, NY, USA).

## Results

**Figure 2:**
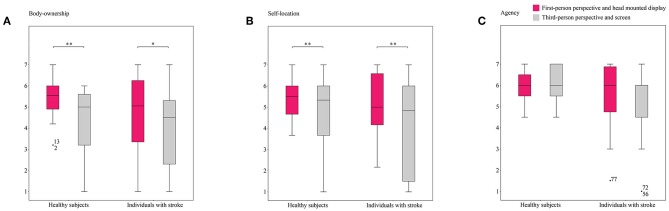
Reports on embodiment sub-constructs in both conditions. The figure shows box and whisker plots of the sense of **(A)** body-ownership, **(B)** self-location, and **(C)** agency experienced by healthy subjects and individuals with stroke in both conditions. *p < 0.05, **p < 0.01.

**Figure 3:**
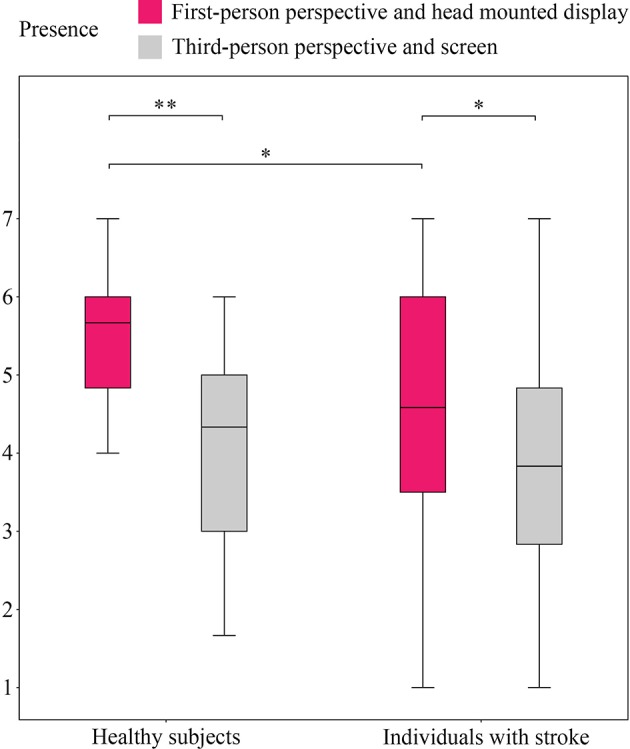
Reports on sense of presence in both conditions. The figure shows a box and whisker plot of the sense of presence experienced by healthy subjects and individuals with stroke in both conditions. *p < 0.05, **p < 0.01.

Scores for the experienced embodiment and presence, and the statistical differences between them, are provided in Figures 2, 3, respectively.

Statistically significant differences were identified between the healthy subjects under the first- and third-person conditions for body-ownership (5.41 ± 0.88 vs. 4.36 ± 1.39; *F*_(1,76)_ = 20.473, *p* < 0.001, $\eta_{\text{p}}^{2}$ = 0.212), self-location (5.43 ± 0.84 vs. 4.74 ± 1.47; *F*_(1,76)_ = 8.553, *p* = 0.005, $\eta_{\text{p}}^{2}$ = 0.101), and presence (5.49 ± 0.81 vs. 4.03 ± 1.24; *F*_(1,76)_ = 50.973, *p* < 0.001, $\eta_{\text{p}}^{2}$ = 0.401). Higher values of these variables were consistently detected for the first-person perspective. Nonetheless, both conditions seemed to successfully induce body-ownership and self-location over the virtual avatar, and presence in the VE, based on the fact that all values exceeded the meaningful threshold. The scores for the sense of presence under the third-person condition were, however, borderline. No differences between conditions were detected for agency (5.98 ± 0.75 vs. 6.04 ± 0.82), which showed the highest values of all the subconstructs of embodiment.

Similar to the healthy participants, statistically significant differences were found between the first- and third-person conditions in the stroke group for body-ownership (4.61 ± 2.02 vs. 3.95 ± 1.92; *F*_(1,76)_ = 5.753, *p* = 0.019, $\eta_{\text{p}}^{2}$ = 0.070), self-location (5.10 ± 1.53 vs. 4.13 ± 2.15; *F*_(1,76)_ = 11.910, *p* = 0.001, $\eta_{\text{p}}^{2}$ = 0.135), and presence (4.45 ± 1.80 vs. 3.83 ± 1.61; *F*_(1,76)_ = 6.357, *p* = 0.014, $\eta_{\text{p}}^{2}$ = 0.077). While these variables were rated as having been vividly experienced in the first-person condition, the third-person condition barely induced self-location over the virtual avatar, and the scores for body-ownership and presence did not (although they almost did) reach the meaningful threshold. As in the healthy group, agency did not show differences between conditions (5.59 ± 1.41 vs. 5.25 ± 1.58), and obtained the highest scores of all the embodiment subconstructs.

A comparison between the groups evidenced statistically significant differences in presence (5.49 ± 0.81 vs. 4.45 ± 1.80; *F*_(1,76)_ = 6.952, *p* = 0.010, $\eta_{\text{p}}^{2}$ = 0.084) in the first-person condition. The scores for embodiment and presence in the healthy group were consistently higher than those in the stroke group for all measures and under both conditions.

## Discussion

This study investigated and compared the sense of embodiment and presence experienced by a sample of healthy subjects and individuals with stroke during exposure to an interactive VR task from different perspectives and under different levels of immersion. The results evidenced that the first-person perspective using HMD elicited a greater sense of body-ownership and self-location over a virtual avatar and a greater sense of presence in both populations. Agency, however, remained almost invariable between conditions. The participants with stroke consistently reported less vivid experiences than the healthy participants, while the differences between the groups were only statistically significant for body-ownership and presence under the first-person condition.

The greater sense of body-ownership and self-location under the first-person condition, and the comparable sense of agency under both conditions, reported by the healthy subjects are in line with previous findings (26, 53–55) and also evidence the distinct natures of the different illusions. The contradictory findings of a previous study, which reported similar vividness of body-ownership and agency, regardless of the perspective, might be explained by a lack of sensitivity in the measurement tools used, as only one question was used to assess each illusion (56). Similar to the effect of perspective on body-ownership and self-location, the greater levels of presence reported by the healthy subjects during the high-immersion condition is supported by previous research (27–29). Interestingly, the only previous experiment that simultaneously investigated embodiment and presence varying the point of view in the VE reported an invariable sense of presence between perspectives for an invariable degree of immersion (26). This could indicate that the differences in presence detected under each condition in our study might be better explained by the difference in immersion rather than perspective. Unfortunately, as both concepts were jointly modified for each condition in our study, it is not possible to identify the determining factor for presence. Despite the differences in the sensed embodiment and presence between conditions, their scores may indicate that healthy subjects are able to successfully incarnate virtual avatars and be present in a virtual world, independent of the perspective and the mediating technology.

Although they did not reach statistical significance, the lower scores for body-ownership and self-location in individuals with stroke in the first-person perspective might suggest a difficulty in these participants to experience the virtual body as their own and being located in it. Although there have been no previous studies that have investigated embodiment in VEs after stroke, experiments involving body illusions in the real world have reported contradictory results that have shown either an increased (43, 57) or decreased (44) predisposition to body-ownership. In our study, post-stroke cognitive disorders, such as a diminished capacity for abstract thinking, which is commonly identified after stroke (58), might have challenged the incarnation of the virtual avatars that, while mimicking the participants' movements, still remained neutral, non-real versions of the participants. In addition, limitations in the body motion-tracking provided by the Kinect v2 (50) and of the mobility of the avatars may have led to some pathological motor patterns in the individuals with stroke to not be exactly reproduced by their virtual selves, which may have reduced their identification of the avatar as their own body and being located in the virtual avatar. This could explain why participants with stroke reported control over the avatar movements, but did not report ownership or self-location. It is important, however, to highlight that the vividness of the embodiment subcomponents was supported by the scores for the different illusions, which exceeded the meaningful threshold under both conditions, but for body-ownership, which were slightly lower than that under the third-person condition. The statistical differences in the sense of presence between the groups were analogous to differences for body-ownership and self-location, with the stroke group showing lower scores. Likewise for these illusions, the cognitive condition of the individuals with stroke might have complicated their interpretations of the VE as real and, consequently, decreased their sense of existing in it; however, also similarly to body-ownership and self-location, scores for this sense support the vividness of the experience.

In summary, the highest scores for sensed body-ownership, self-location, and presence, were provided by the healthy subjects, regardless of condition, and for the first-person perspective with a HMD, regardless of the group. The sense of agency, in contrast, received invariably high scores under all conditions and for both groups. The analogous scores for individuals with stroke and the healthy control group support that the sense of embodiment and presence were similarly experienced in both populations. This suggests that the basic mechanisms that modulate these phenomena could be preserved after a stroke, and may support the effectiveness of VR interventions in this population.

## Conclusions

A sense of embodiment and presence were effectively experienced in both healthy subjects and individuals post-stroke, although less intensively in the latter. The feelings were similarly modulated by perspective and level of immersion.

## Data Availability Statement

The datasets generated for this study will not be made publicly available confidential data.

## Ethics Statement

Ethical approval for the study was granted by the Institutional Review Board of Vithas Hospital Valencia al Mar (TU763198CIS0/1). All participants provided written informed consent before taking part in the study.

## Author Contributions

AB and RL designed the study. RL defined the clinical aspects regarding individuals with stroke and JL assessed their condition. AB and JL conducted the experimental sessions. All the authors analyzed and discussed the results of the experiment.

### Conflict of Interest

The authors declare that the research was conducted in the absence of any commercial or financial relationships that could be construed as a potential conflict of interest.
